# The retrotransposon R2 maintains *Drosophila* ribosomal DNA repeats

**DOI:** 10.1073/pnas.2221613120

**Published:** 2023-05-30

**Authors:** Jonathan O. Nelson, Alyssa Slicko, Yukiko M. Yamashita

**Affiliations:** ^a^Whitehead Institute for Biomedical Research, Cambridge, MA 02142; ^b^HHMI, Cambridge, MA 02142; ^c^Department of Biology, School of Science, Massachusetts Institute of Technology, Cambridge, MA 02142

**Keywords:** ribosomal DNA, retrotransposons, Drosophila, germline

## Abstract

Ribosomal DNA (rDNA) loci contain hundreds of tandemly repeated copies of ribosomal RNA genes needed to support cellular viability. This repetitiveness makes it highly susceptible to copy number (CN) loss due to intrachromatid recombination between rDNA copies, threatening multigenerational maintenance of rDNA. How this threat is counteracted to avoid extinction of the lineage has remained unclear. Here, we show that the rDNA-specific retrotransposon R2 is essential for restorative rDNA CN expansion to maintain rDNA loci in the Drosophila male germline. The depletion of R2 led to defective rDNA CN maintenance, causing a decline in fecundity over generations and eventual extinction. We find that double-stranded DNA breaks created by the R2 endonuclease, a feature of R2’s rDNA-specific retrotransposition, initiate the process of rDNA CN recovery, which relies on homology-dependent repair of the DNA break at rDNA copies. This study reveals that an active retrotransposon provides an essential function for its host, contrary to transposable elements’ reputation as entirely selfish. These findings suggest that benefiting host fitness can be an effective selective advantage for transposable elements to offset their threat to the host, which may contribute to retrotransposons’ widespread success throughout taxa.

## Significance

Retrotransposons are mobile genetic elements that occupy a large fraction of eukaryotic genomes, but are generally regarded as genomic parasites that do not contribute to host biology. This study reveals that the Drosophila retrotransposon R2 has a function essential to maintain its hosts genome. R2 specifically mobilizes within the large number of tandem ribosomal DNA (rDNA) repeats needed for proper ribosomal function. rDNA repeats are frequently lost from the genome, and restoration of these lost copies within the germline is required to maintain rDNA over successive generations. We find that R2 activity stimulates the expansion of rDNA copies needed to maintain rDNA throughout a population, indicating that R2 mutually benefits the survival of its host genome and its own propagation.

**Fig. 1.**
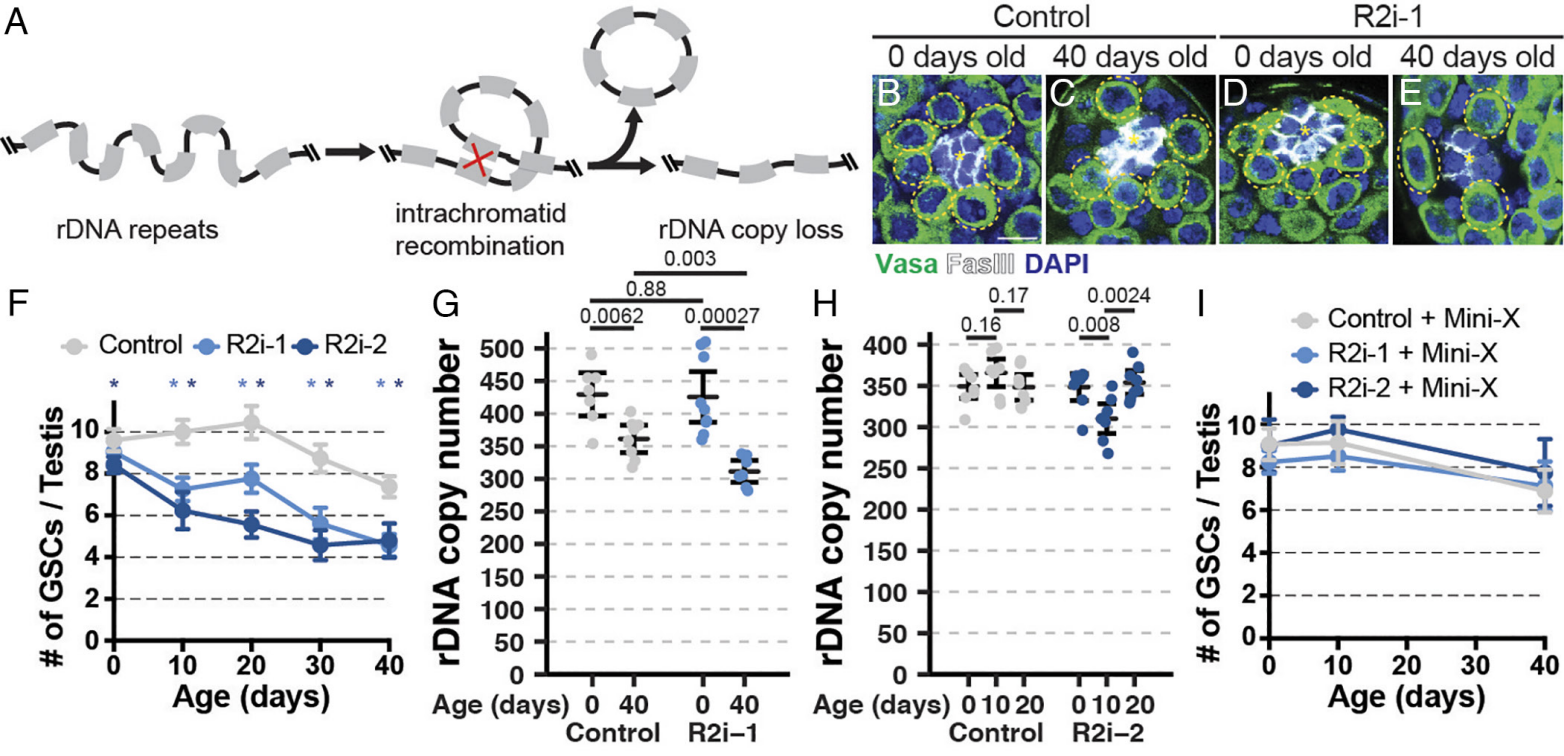
R2 is required for GSC maintenance via rDNA CN maintenance during Drosophila male germline aging. (*A*) Model of rDNA repeat instability. (*B*–*E*) GSCs in 0- and 40-d old control (*B* and *C*) and R2 RNAi testes (*D* and *E*). Yellow dotted circle = GSC. GSC signaling niche indicated by *. Green = Vasa, White = Fascillin III, Blue = DAPI. (Scale bar, 7.5 µM). (*F*) Average GSCs per testis in control and two R2 RNAi lines during aging. * indicates *P* < 10^−3^ determined by Student’s *t* test. Error = 95% CI. (*G* and *H*) Testis rDNA CN determined by ddPCR. *P* value by Student’s *t* test. Error = 95% CI. (*I*) Average GSCs per testis during aging in Control and two R2 RNAi lines containing mini-X chromosome. Error = 95% CI.

Ribosomal RNAs (rRNAs) account for 80 to 90% of all transcripts in eukaryotic cells (1). To meet this demand, the ribosomal DNA (rDNA) gene that codes for rRNA is tandemly repeated hundreds of times, making up rDNA loci on eukaryotic chromosomes. This repetitive structure is susceptible to intrachromatid recombination that causes rDNA copy number (CN) loss (Fig. 1*A*), which is a major cause of replicative senescence in budding yeast (2). Evidence of similar rDNA CN instability has been noted in some tissues from aged dogs and humans (3, 4). While rDNA instability in somatic tissues may lead to insufficient ribosomal activity and disrupt cellular function, perhaps leading to disease and threatening the health of the individual (5), rDNA instability in the germline threatens survival of the entire species due to the potential degeneration of rDNA loci over successive generations. rDNA CN is variable between individuals of most species but maintained within a consistent range throughout the population (6), implying that rDNA CN is dynamically maintained through transgenerational series of CN losses and re-expansions. Indeed, age-associated rDNA CN loss occurs in the *Drosophila* male germline and is inherited by the next generation, but the flies that inherited reduced rDNA CN re-expand rDNA in their germline to ensure sufficient rDNA is transmitted to their progeny (7). Similarly, intensive studies have revealed that rDNA CN expansion in yeast maintains rDNA repeat abundance over generations through sister chromatid recombination at rDNA loci (2).

Germline rDNA CN restoration has been best studied in Drosophila, particularly in the phenomenon of “rDNA magnification,” first described over 50 years ago as the process wherein aberrant rDNA loci bearing minimal rDNA repeats recover to a normal rDNA CN (8, 9). rDNA magnification is proposed to be accomplished through unequal sister chromatid exchange (USCE) during the homologous recombination (HR)-mediated repair of double-stranded breaks (DSBs) at the rDNA locus (10). This proposed USCE mechanisms is similar to the model of tandem rDNA repeat expansion in yeast (11), except that it results in one sister chromatid gaining rDNA copies at the cost of “stealing” them from the other sister. Indeed, rDNA magnification requires genes involved in homologous recombination (HR)-mediated repair (12, 13), supporting the model of USCE-mediated rDNA CN expansion. We have demonstrated that the expansion of rDNA CN in the germline of the progeny from old fathers requires the same set of genes as rDNA magnification (7), suggesting that the mechanisms of rDNA magnification normally serve to maintain rDNA CN across generations. This rDNA CN expansion likely operates in male germline stem cells (GSCs) (7), which support sperm production throughout adulthood through persistent asymmetric divisions that produce a self-rendered GSC and a differentiating daughter destined for sperm differentiation (14). We recently found that GSC divisions have biased inheritance of sister chromatids that preferentially retains the sister chromatid with more rDNA copies in the GSC during rDNA CN expansion, leading us to propose that USCE followed by retention of the expanded rDNA locus in the GSC achieves rDNA magnification (15). While these observations may explain how rDNA CN expansion occurs in the germline, the underlying factors that control this process, particularly the source of DNA breaks at rDNA loci that may initiate USCE, remain elusive.

Metazoan rDNA genes frequently contain insertions of rDNA-specific transposable elements (TEs), such as the retrotransposon R2 in Drosophila. R2 is found throughout arthropods and R2-like elements are widely present across taxa, including Cnidaria, Planaria, nematodes, fish, birds, and reptiles (16, 17). These TEs use their sequence-specific nuclease to mobilize specifically within rDNA loci (18), inserting into rDNA genes and likely disrupting 28S rRNA function (19) (*SI Appendix*, Fig. S1*A*). TEs are generally regarded as genomic parasites, serving only their replication throughout the host genome via their mobilization, which typically has detrimental, or at best neutral, mutagenetic effects. Many TEs mitigate their potential detriment to the host through biased mobilization at “harmless” insertion sites, such as repetitive, noncoding, or heterochromatic regions (20). Here we show that the Drosophila R2 retrotransposon actively contributes to host functions needed for rDNA CN expansion: inhibition of R2 expression in the germline disrupts rDNA maintenance, resulting in GSC loss and reduced fecundity over successive generations. We find that the R2 rDNA-specific endonuclease is required for R2 to induce rDNA CN expansion, indicating that R2 activity is the source of DSBs at rDNA that can stimulate USCE. We propose that R2 is a “mutualistic” TE whose mobilization activity benefits host fitness, which in turn benefits their own evolutionary success.

## Results

### R2 Is Required for Normal Germline rDNA CN and GSC Maintenance during Aging.

To test the potential impact of R2 in the Drosophila male germline, we conducted RNAi-mediated knockdown of R2 in the Drosophila male germline (*nos-gal4*-driven expression of RNAi lines, *nos*>*R2i-1* or *R2i-2,* hereafter) (*SI Appendix*, Fig. S1*A*). RNAi constructs were specifically designed to target the open reading frame (ORF) part of R2, which is included in the mature R2 RNA that is translated to produce the sequence-specific endonuclease/reverse transcriptase protein that executes R2 retrotransposition (19, 21). Because translation of R2 ORF occurs in the cytoplasm, the RNAi machinery (which mainly operates in the cytoplasm) is expected to efficiently target R2 ORF expression. Indeed, we observed R2 transcripts are efficiently knocked down by expression of these RNAi constructs, without disrupting expression of the rDNA arrays where they are inserted (*SI Appendix*, Fig. S1 *B*–*H*). Since R2 is a multicopy TE, we could not achieve complete elimination of R2 transcripts, but our knockdown was similarly efficient to previously reported use of RNAi-mediated knockdown of TE transcripts that functionally disrupted TE activity (22). Surprisingly, we found that RNAi-mediated knockdown of R2 resulted in premature loss of GSCs during aging (Fig. 1 *B*–*F*). GSCs continuously produce differentiating germ cells to sustain sperm production throughout adulthood and thus are the source of the genome passed to the next generation (14). Whereas newly eclosed R2 RNAi males contained similar numbers of GSCs to controls, GSC number more rapidly declined during aging in R2 knockdown males compared to controls (Fig. 1*F*). Given that R2 is specifically inserted into rDNA, we also examined the effect of R2 knockdown on rDNA stability. Using highly quantitative droplet digital PCR (ddPCR), we found that while RNAi-mediated knockdown of R2 had no effect in young testes, older RNAi expressing animals had reduced rDNA copy number compared to controls (Fig. 1 *G* and *H*), suggesting rDNA CN loss is more severe during aging when R2 is inhibited. rDNA CN loss in the germline was further confirmed by DNA FISH on the meiotic chromosomes (*SI Appendix*, Fig. S2 *A*–*F*). Interestingly, one of the RNAi constructs (*R2i-2*) suffered rapid rDNA CN loss within the first 10 d of adulthood, but recovered by 20 d of age (Fig. 1*G*). This effect may be due to the incomplete efficiency of RNAi knockdown, combined with the variable multicopy nature of R2 within rDNA, which appears to leave a small population of germ cells that retain the ability to express R2 in the presence of the RNAi (*SI Appendix*, Fig. S1*B*). We speculate that severe rDNA loss caused by the *R2i-2* RNAi may rapidly select for such germ cells, which become enriched within the testis, accounting for the observed recovery in rDNA CN at later ages. rDNA CN insufficiency is likely the primary cause of GSC loss in R2 RNAi animals, because increasing total rDNA CN via introduction of a mini-chromosome harboring an rDNA locus (23) suppressed the premature GSC loss caused by R2 knockdown (Fig. 1*I*). These results revealed that R2 contributes to sustaining GSC population during aging through rDNA CN maintenance, uncovering an unanticipated benefit of the R2 retrotransposon to the host, despite the widely held view of mobile TEs being genetic parasites.

### R2 Is Necessary and Sufficient for rDNA Magnification.

**Fig. 2.**
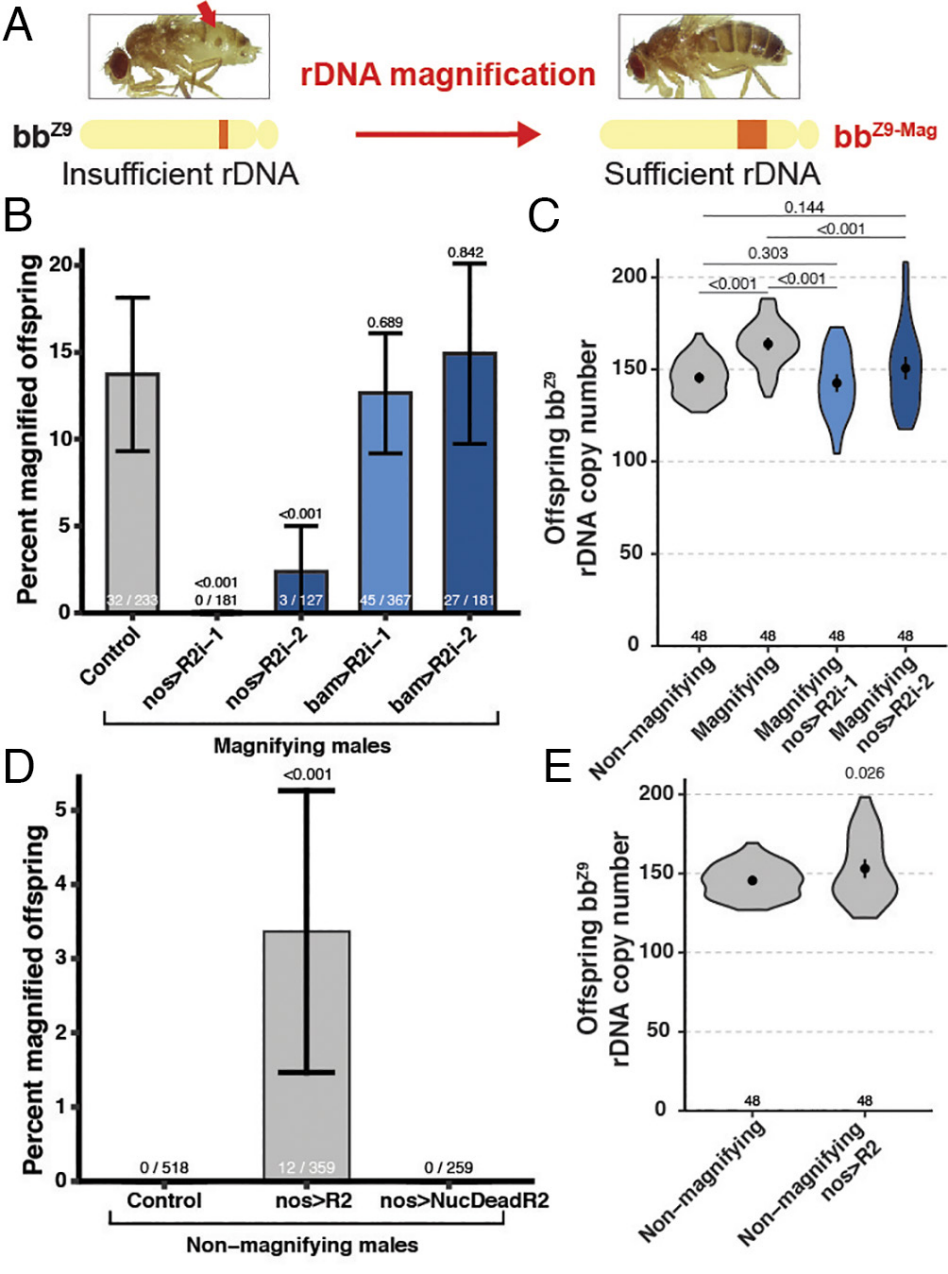
R2 is necessary and sufficient for rDNA magnification. (*A*) Diagram of rDNA magnification at the *bb^Z9^* rDNA locus, during which dorsal cuticle defect (red arrow) revert to normal cuticle. (*B*) Percent magnified offspring determined by cuticular phenotype in offspring from magnifying males. *P* value determined by chi-squared test. Error = 95% CI. (*C*) Mean *bb^Z9^* locus rDNA CN determined by ddPCR in daughters from males. *P* value determined by Student’s *t* test. Error = 95% CI. (*D*) Percent magnified offspring from nonmagnifying males. *P* value determined by chi-squared test. Error = 95% CI. (*E*) Mean *bb^Z9^* locus rDNA CN determined by ddPCR in daughters from nonmagnifying males. Nonmagnifying condition is the same data as panel *C*. *P* value determined by Student’s *t* test. Error = 95% CI. For all experiments, control condition contains *nos-gal4* alone with no RNAi or transgene.

Historically, rDNA magnification has been assessed through the observation of the emergence of offspring with normal cuticle from fathers with abnormal (“bobbed”) cuticle caused by insufficient rDNA CN (8) (Fig. 2*A*). *Drosophila melanogaster* rDNA loci reside on the sex chromosomes (X and Y) (24), and rDNA magnification is typically assayed as the recovery of X chromosome rDNA CN: X chromosome rDNA loci harboring the minimal viable amount of rDNA (*bb^Z9^,* *SI Appendix*, Fig. S3*A*) undergoes magnification when combined with a Y chromosome lacking rDNA (*bb^Z9^/Ybb^0^*, “magnifying males” hereafter) (8) (*SI Appendix*, Fig. S3*B*). Importantly, the use of *Ybb^0^* is required to induce rDNA magnification, presumably because a normal Y chromosome provides sufficient rDNA copy number (and thus does not activate the CN sensing mechanism to trigger magnification). Accordingly, rDNA magnification is typically not observed in males with a normal Y chromosome containing intact rDNA (*bb^Z9^/Y^+^*, “nonmagnifying males” hereafter) (9). We found that R2 knockdown reduces rDNA magnification from 13.73% in control conditions (*bb^Z9^/Ybb^0^; nos-gal4*, n = 233) to 0% in R2i-1 (*bb^Z9^/Ybb^0^; nos-gal4/UAS-R2i-1*, n = 181, *P* = 5.6 × 10^−7^) and 2.36% in R2i-2 animals (*bb^Z9^/Ybb^0^; nos-gal4/UAS-R2i-2*, n = 127, *P* = 9.9 × 10^−4^) (Fig. 2*B*). Moreover, the quantification of rDNA CN by ddPCR revealed that 87.5% of *bb^Z9^* chromosomes increased rDNA CN in magnifying males (n = 96, *P* = 1.8 × 10^−4^), with an average increase of 18.29 rDNA copies across all samples (n = 96, *P* = 3.1 × 10^−12^) (Fig. 2*C*). Importantly, this observed increase in rDNA CN under magnifying conditions was detected from animals randomly selected across all offspring, including those that still had *bobbed* cuticle phenotype: this demonstrates that rDNA magnification broadly increases rDNA CN throughout the germline, despite only 13.73% of *bb^Z9^* chromosomes recovering enough CN to support normal cuticle development. This rDNA CN increase in magnifying males is also eliminated upon R2 knockdown (Fig. 2*C*). These results reveal that R2 is required for rDNA CN expansion during rDNA magnification. Interestingly, we found that rDNA magnification was blocked only when the R2 RNAi constructs were expressed by the *nos*-*gal4* driver in early germ cells (including GSCs), but not when expressed in later germ cells by the *bam*-*gal4* driver (*Materials and Methods*) (Fig. 2*B*). These results indicate that R2 primarily functions in the earliest stages of germ cells (including GSCs) to support rDNA magnification.

We further found that R2 is sufficient for rDNA CN expansion. Ectopic expression of transgenic R2 in the germline (*SI Appendix*, Fig. S4 *A*–*F*) induced rDNA magnification of the *bb^Z9^* locus in nonmagnifying males (*bb^Z9^/Y^ +^*) (Fig. 2 *D* and *E*). We found 3.3% of female offspring from males expressing transgenic R2 (*bb^Z9^/Y^+^; UAS-R2/+; nos-gal4/+*) exhibited magnification (normal cuticle) (Fig. 2*D*, n = 877, *P* = 3.2 × 10^–5^), compared to control (*bb^Z9^/Y^+^*; *nos-gal4* without R2 expression), which never showed any magnification, when dorsal cuticle phenotype was used as an assay. Thus, transgenic R2 expression is sufficient to induce rDNA magnification, albeit at a low frequency. Importantly, reversion of the cuticle phenotype was heritable to the subsequent F2 generation throughout our experiments, confirming that CN restoration occurred in the germline (*SI Appendix*, Fig. S5 *A*–*C*). Quantification of rDNA CN by ddPCR revealed that ectopic overexpression of R2 in nonmagnifying males (*bb^Z9^/Y^+^*) also increases the average rDNA CN at *bb^Z9^* rDNA loci among all offspring, again regardless of cuticular phenotype (Fig. 2*E*, n = 94, *P* = 0.0256), revealing R2 expression induces rDNA CN expansion broadly among inherited rDNA loci. Critically, expression of a nuclease dead R2 transgene (NucDeadR2) in nonmagnifying males (*SI Appendix*, Fig. S4 *A*–*E*) failed to induce rDNA magnification (Fig. 2*D*), suggesting that the nuclease activity of R2 is essential for its ability to induce rDNA CN expansion.

### The R2 Endonuclease Is Required to Induce rDNA CN Expansion.

**Fig. 3.**
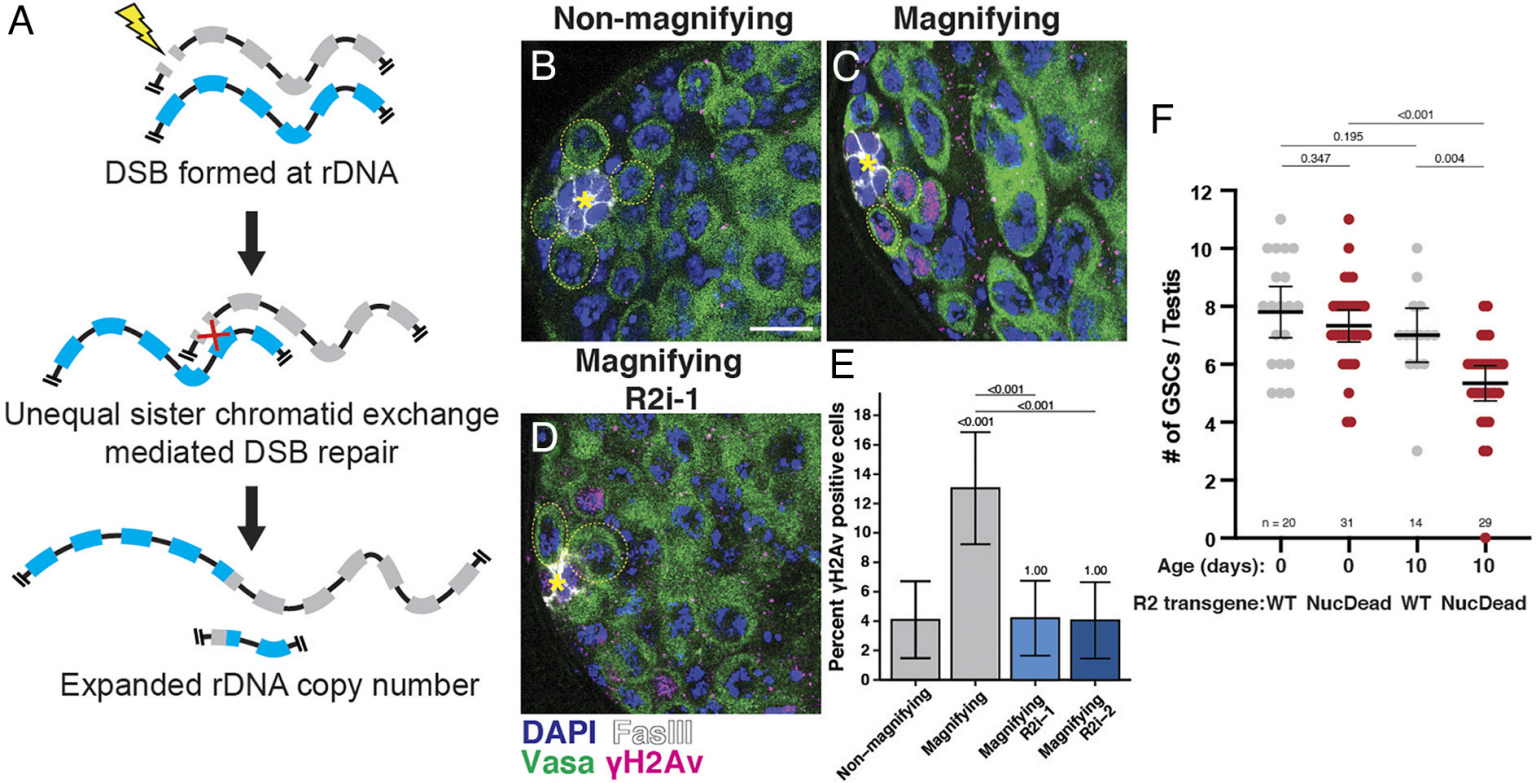
Derepressed R2 creates DSBs in GSCs during rDNA magnification. (*A*) Diagram of rDNA CN expansion by unequal sister chromatid exchange during DSB repair at rDNA loci. Recombination between misaligned rDNA copies during DSB repair result in crossovers that create unequal sister chromatid exchange that increases rDNA CN on one chromatid. (*B*–*D*) Detection of DSBs in the early adult male germline by anti-γH2Av staining. R2 RNAi expressed under the *nos-gal4* driver. Non-RNAi conditions contain the *nos-gal4* driver alone. GSCs indicated by yellow dotted circle. Blue = DAPI, Green = vasa, Magenta = γH2Av, white = FasIII. The hub is indicated by *. (Scale bar, 10 µM). (*E*) Percentage of γH2Av positive GSCs. *P* value determined by chi-squared test. Error = 95% CI. (*F*) Number of GSCs per testis in R2i-1 condition coexpressing R2 transgenes. *P* value determined by Student’s *t* test. Error = 95% CI.

In yeast, rDNA CN expansion is initiated by DSBs at the rDNA intergenic sequence, which induces sister chromatid recombination that results in rDNA gene duplication (25). All proposed models of Drosophila rDNA CN expansion [the most prominent model being USCE (10)] require an initiating DSB at the rDNA locus (Fig. 3*A* and *SI Appendix*, Fig. S6 *A* and *B*). Indeed, artificial introduction of DSBs at rDNA loci by I-CreI endonuclease expression has been reported to induce rDNA magnification (26), but the endogenous factor that induces rDNA magnification remained unclear. R2 is capable of creating DSBs through sequential nicking of opposite DNA strands during retrotransposition (16). It has been speculated that DSBs created during R2 retrotransposition may be an initiating event of rDNA magnification (27), although this possibility has yet to be empirically tested. We found that rDNA magnification is associated with an elevation in DSBs in GSCs: the frequency of γH2Av-positive GSCs is increased in magnifying males (*bb^Z9^/Ybb^0^*) compared to nonmagnifying males (*bb^Z9^/Y^ +^*) (Fig. 3 *B,* *C*, and *E*; n = 519, *P* = 8.8 × 10^−4^). Strikingly, we observed that knockdown of R2 in magnifying males reduced the frequency of γH2Av-positive GSCs to levels comparable to nonmagnifying males (Fig. 3 *D* and *E*; n = 537, *P* = 7.1 × 10^−4^ for *R2i-1*; n = 521, *P* = 7.9 × 10^−4^ for *R2i-2*), indicating that R2 is responsible for the DSBs formed in GSCs during rDNA magnification. Furthermore, we confirmed that expression of transgenic R2, but not NucDeadR2, indeed induces chromosomal breaks at rDNA loci identified by chromosome spreads (*SI Appendix*, Fig. S4 *B–D*). R2 overexpression (but not NucDeadR2) in the germline also increased the frequency of GSCs with DSBs, identified by γH2Av expression (*SI Appendix*, Fig. S4*E*). Taken together, these results suggest that rDNA-specific endonuclease activity of R2 creates DSBs at the rDNA loci that may in turn induce rDNA CN expansion. Importantly, R2 transgenes (UAS-R2 and UAS-R2NucDead) contain synonymous mutations that confer resistance to the R2i-1 RNAi, and the expression of functional R2 but not NucDeadR2 was able to rescue the disruption of GSC homeostasis caused by R2 RNAi (*SI Appendix*, Fig. S4*F*). These findings confirm that the defects in GSC homeostasis upon R2 RNAi expression are indeed due to loss of R2 function, and suggest that R2 endonuclease activity is required for R2 contribution to rDNA maintenance in GSCs.

### R2 Is Dynamically Regulated within GSCs in Response to rDNA CN.

**Fig. 4.**
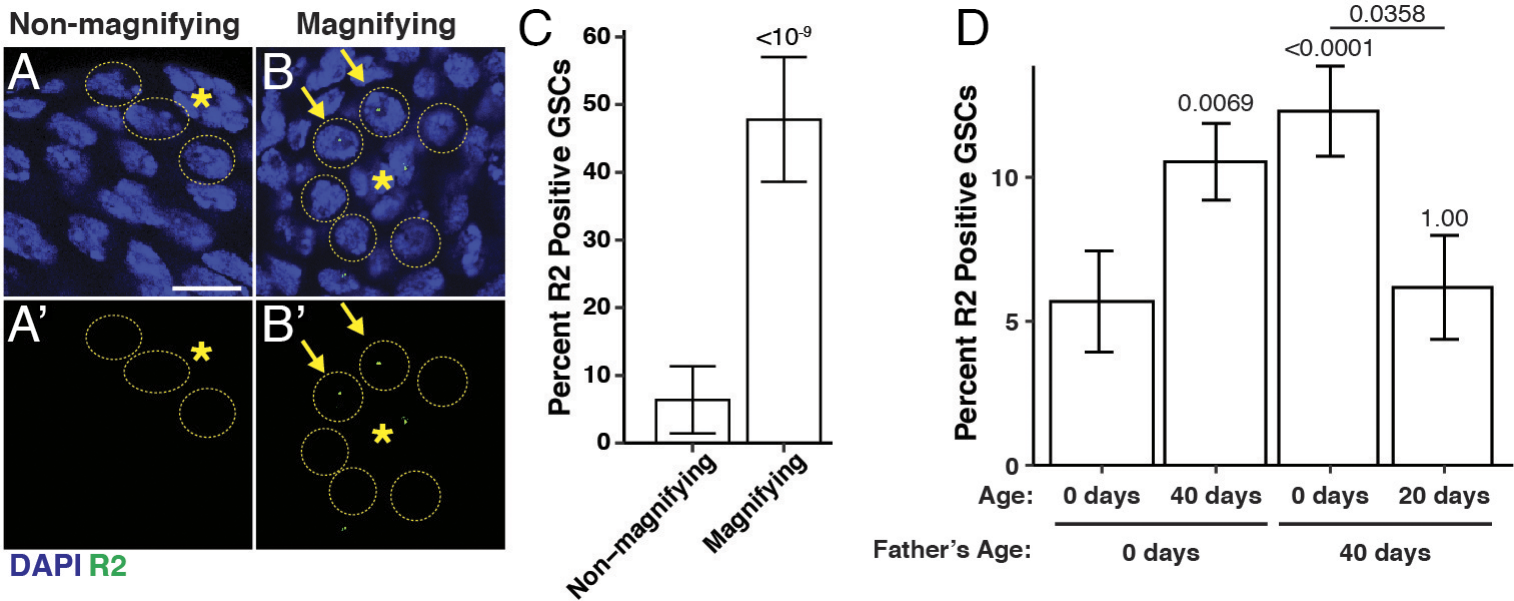
R2 expression is regulated in response to changes in rDNA copy number. (*A* and *B*) R2 expression in GSCs (yellow dotted circle). Blue = DAPI, Green = R2 mRNA. Isolated R2 channel in A′ and B′. The hub is indicated by *. R2 positive cells GSCs are marked by yellow arrowhead. (*C*) Percentage of R2 positive GSCs in nonmagnifying (*Y^+^/bb^Z9^*) and magnifying (*Ybb^0^/bb^Z9^)* animals. (*D*) Percentage of R2 positive GSCs in newly eclosed and aged adults from young or old fathers. *P* values determined by chi-squared test. Error = 95% CI.

Given the threat R2 mobilization poses to the host genome, both by disruption of rRNA function and causing excessive DSB formation (16), how is the potential benefit of R2 to rDNA CN maintenance balanced with the detriment of R2 retrotransposition? We found R2 expression in the germline is specifically derepressed under conditions of reduced rDNA CN, potentially explaining how the conflicting consequences of R2 expression are resolved. Using RNA fluorescence in situ hybridization (RNA FISH) to examine R2 expression at a single-cell resolution, we found that the frequency of GSCs expressing R2 was significantly increased in magnifying males (*bb^Z9^/Ybb^0^*), whereas nonmagnifying males (*bb^Z9^/Y^+^)* rarely expressed R2 (Fig. 4 *A*–*C*; n = 231, *P* = 1.7 × 10^−10^). Moreover, we found that GSCs from aged animals and the sons of old fathers, which inherit reduced rDNA CN (7), also exhibited a higher frequency of R2 expression compared to GSCs from young flies (Fig. 4*D* and *SI Appendix*, Fig. S7 *A* and *B*; n = 1,247, *P* = 8.3 × 10^−4^ for old animals; n = 1,107, *P* = 1.5 × 10^−4^ for offspring). Importantly, the frequency of R2 expression among GSCs in the sons of old fathers returned to the basal level after 20 d of age, when rDNA CN was shown to have recovered (7) (Fig. 4*D* and *SI Appendix*, Fig. S7*C*; n = 617, *P* = 0.036). These results indicate that R2 expression is dynamically regulated in response to changing rDNA CN. Taken together, we propose that R2 expression is finely tuned to function when most beneficial to the host while minimizing unnecessary exposure to the harmful effects of transposition.

### R2-Mediated rDNA CN Expansion Is Required for Multigenerational Maintenance of Germline Function.

**Fig. 5.**
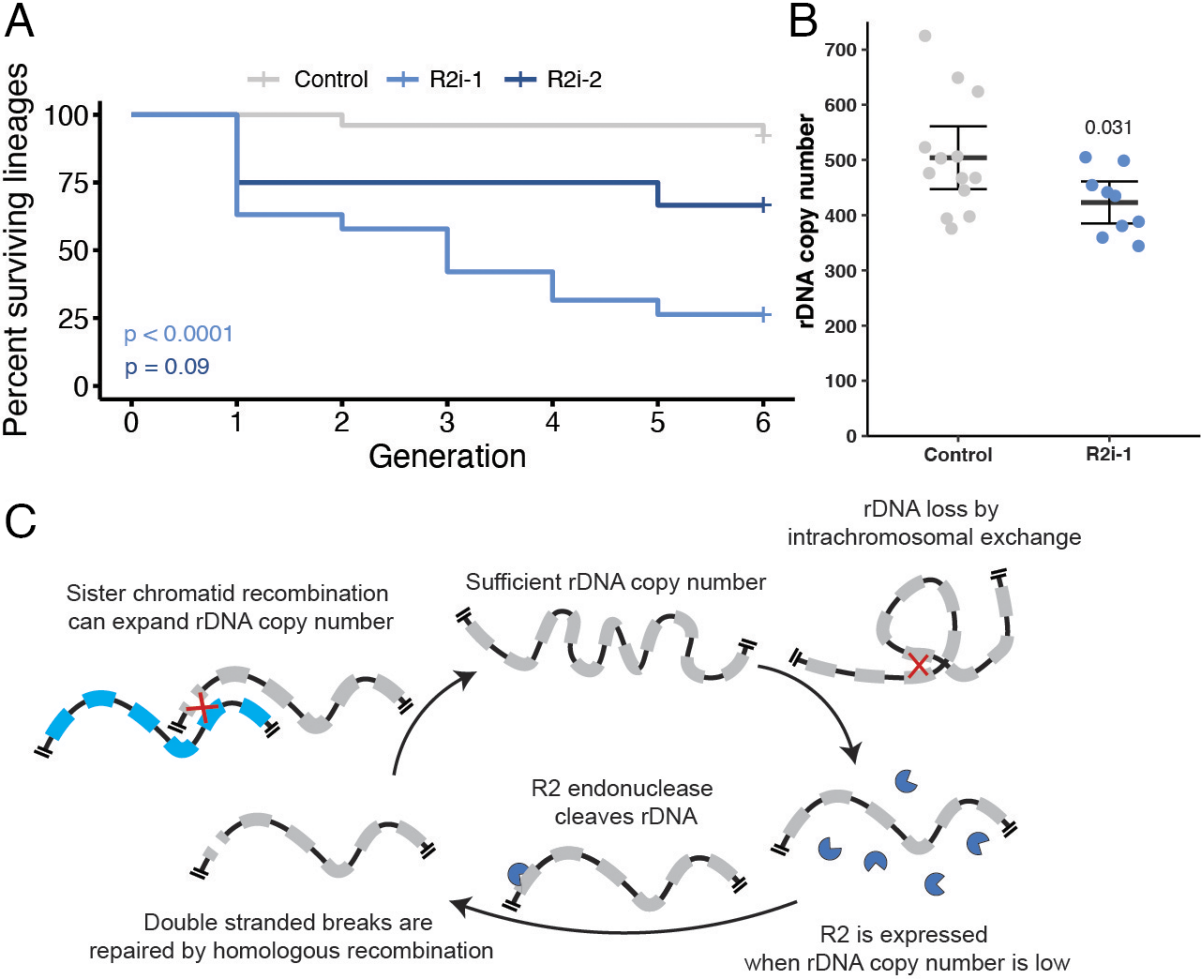
R2 is required to maintain rDNA CN and fertility over successive generations. (*A*) Kaplan–Meier curve of lineage survival in control (*nos-gal4* driver alone and two R2 RNAi expressing via the *nos-gal4* driver) lineages. Each lineage constitutes an individual data point. *P* values determined by log rank test. (*B*) rDNA CN determined by ddPCR in males of control animals at the 6th generation or R2 RNAi lineages at their terminating generation. *P* value determined by Student’s *t* test. Error = 95% CI. (*C*) Model of the role of R2 in germline rDNA CN maintenance.

Based on the finding that R2 plays a critical role in maintaining germline rDNA CN, we postulated that R2 is essential to prevent continuous multigenerational rDNA loss capable of causing the extinction of the lineage. In *C. elegans*, the loss of genome integrity is known to cause gradual loss of fertility, a phenotype known as mortal germline (morg) (28). To test whether R2-mediated rDNA maintenance is required to maintain fertility through generations, we established multiple independent lines expressing R2 RNAi in their germline and tracked their fecundity at each generation through the ability of each line to produce sufficient offspring to establish a new generation (*SI Appendix*, Fig. S8). While nearly all control lines survived throughout the duration of the experiments, we found that lines expressing the *R2i-1* RNAi construct failed to consistently produce sufficient progeny, with over half failing by the fourth generation (Fig. 5*A*) (n = 43, *P* = 3 × 10^−6^), indicating that R2 is essential for continuity of the germline lineage. Surviving males of extinguishing *R2i-1* lineages had ~20% reduction in rDNA CN compared to control lines (n = 22, *P* = 0.031) (Fig. 5*B*). With the *R2i-2* RNAi, the lineage was maintained relatively well, after initial sharp drop (Fig. 5*A*): Considering that R2 knockdown by the *R2i-2* construct exhibits only transient rDNA CN decrease at day 10 (*SI Appendix*, Fig. S2*A*), which we speculate is produced through selection of germ cells that have sporadically retained R2 expression in the RNAi condition, this effect may also quickly select for lineages insensitive to R2 knockdown that have retained rDNA CN expansion activity. Taken together, these results suggest that R2-mediated maintenance of rDNA contributes to germline immortality.

## Discussion

Our findings reveal an unanticipated “function” of retrotransposon activity to benefit the host genome through a role in rDNA CN maintenance. Tandem repetitive DNA sequences are among the elements of the eukaryotic genome most vulnerable to genomic excision (29), and essential tandemly repeated coding and noncoding elements likely require active mechanisms that serve their maintenance. We propose that rDNA loci are maintained by DSBs generated by R2 in GSCs with reduced rDNA CN, which stimulate sister chromatid exchange that results in rDNA CN expansion to restore CN (Fig. 5*C*). We recently reported that USCE in GSCs followed by nonrandom segregation of the “expanded” sister chromatid to the self-renewed GSC during mitosis likely mediates rDNA magnification (15). The present study suggests that R2 functions upstream of USCE, creating the DSBs that stimulate this repeat expansion mechanism, revealing the function that TEs plays for the host. This work provides experimental evidence that supports the model for R2 to initiate rDNA magnification first suggested over 30 years ago by Hawley and Marcus (27), which was not possible to test at the time in the absence of methods to inhibit the expression of multicopy TEs (e.g., RNAi).

TEs can be a major source of genomic instability, generating DNA breaks, disrupting genes as they mobilize, or creating opportunities for recombination between TE insertions (30). There is often selective advantage for TEs to minimize the threat of their mobilization through restricting their expression or limiting their range of mobilization (20). Our proposed role for R2 in rDNA maintenance suggests that contributing to host functions may also be a robust adaptive feature for TEs through creating a mutualistic host–TE relationship. Since DSB formation by the R2 endonuclease relies on reverse transcriptase activity (18), it remains unclear whether full integration of a new R2 insertion or DSB generation alone stimulates rDNA CN expansion. Further analysis that can separate R2 endonuclease activity from reverse transcription will reveal if R2 contributes to rDNA CN expansion beyond DSB formation alone. There are several descriptions of TEs providing benefit to their host through their coding or noncoding features being repurposed for host function, but these “co-opted” TEs lack their own ability to mobilize and replicate (31). There are very few well-described examples of eukaryotic “mutualistic” TEs, whose active mobilization benefits host fitness (32). The only other functionally demonstrated mutualistic TEs are the telomere-bearing element (TBE) DNA transposases in the ciliate *Oxytricha trifallax* (33). The TBEs execute the large-scale genome rearrangement needed for *O. trifallax* macronuclear development, and knockdown of TBE transposons by RNAi disrupts macronuclear assembly (33). The first proposed “functional” TEs are the retrotransposons that constitute the telomeres of most Drosophila species (Het-A, TART, TAHRE) (34). The absence of telomerase from these species, combined with evidence that suggests their transposition may be licensed by the host, lead to the model that retrotransposition of these telomeric TEs maintains telomeres in the fly (35). Although retrotransposition of telomeric TEs is indeed required to establish new telomeres at broken chromosome ends (36), it has yet to be functionally demonstrated whether these TEs are required to maintain existing telomeres. On the contrary, recombination-based, TE-independent telomere extension has been observed to be a major source of telomere extension in Drosophila (37) and some Drosophila species completely lack functional telomeric TEs, appearing to rely solely on this recombination-based telomere extension (38). Therefore, while telomeric TEs may potentially be mutualistic elements, their requirement for telomere maintenance and host fitness remains unclear, and these elements may have instead simply found a safe-haven for insertion at telomeres. There appears to be a fine line between mutualistic element and opportunistic parasite, and further discovery of functional instances of mutualistic TEs will be critical to understand how host–TE relationships may shift between parasitism and mutualism.

For the mutualistic host–TE relationship to exist, R2 expression is likely dynamically regulated through the interaction with the host, such that its expression is limited to only when it can be beneficial (i.e., decreased rDNA CN). This control may be achieved through modulation of a number of mechanisms that can regulate R2 expression. The piRNA pathway is the major repressor of TEs in the germline, including R2 (39). Indeed, R2 is normally repressed in the male germline, but becomes derepressed only when rDNA CN becomes insufficient (Fig. 4 *A*–*C*). Thus, it is possible that the activity of the piRNA pathway is modulated such that R2 becomes derepressed only when germ cells experience insufficient rDNA CN (e.g., changes in the activity of piRNA core machinery or a reduction in R2-specific piRNAs). It is also possible that the transcription of R2 may be regulated. Lacking its own Pol II promoter, R2 transcription is dependent on transcription of the rDNA copy where it is inserted (40), suggesting changes in rDNA transcription upon rDNA CN loss may alter R2 expression. Indeed, we observed large-scale transcriptional changes occur at rDNA loci in GSCs upon rDNA CN loss (7), suggesting regulation over rDNA transcription may control R2 expression in response to rDNA CN loss. rDNA transcription and stability are broadly impacted by siRNA-mediated histone methylation (41, 42), and modification of this activity may underlie the increased R2 expression upon rDNA reduction. Furthermore, disruption of ribosome processing has also been shown to selectively induce R2 expression (43). This indicates that ribosomal abundance or function may be the molecular sensor that triggers R2 expression upon rDNA CN reduction, perhaps through a compensatory activation of hitherto repressed rDNA copies, including those containing R2. Future investigation to uncover how these mechanisms may control R2 expression in response to rDNA CN are critical to understanding how R2 can be utilized for the host’s benefit.

The elucidation of the nature of the rDNA CN sensing mechanism is critical to understand how R2 expression is regulated in response to rDNA CN. Curiously, in our R2 RNAi conditions we observed robust defects in germ line viability and function while detecting modest deficiencies in rDNA CN itself, suggesting R2 expression and rDNA CN expansion in the germline may be triggered by relatively small reductions in rDNA CN. This modest effect may be partly due to survivorship bias in our sampling, since rDNA CN cannot be measured in nonviable animals that have fewer rDNA copies than required for viability. Additionally, the phenotypic effects of these small CN changes may be due to a physiological need to maintain large rDNA CN beyond the small subset needed for transcription at any given time (44). rDNA loci are a common site of transcription–replication collisions that can create DNA breaks when replication forks progress through highly transcriptionally active regions of the rDNA (45). Accordingly, cells must compartmentalize rDNA copies into those that are actively transcribed and others that are being replicated to avoid transcription–replication collisions. Because of the need of compartmentalization, cells must carry more rDNA CN than minimally required to transcribe sufficient rRNA for cells’ survival. The transcription–replication collisions become more likely to occur as rDNA loci shrink, and their increased frequency compromises the efficiency of DNA damage repair activity (2). Indeed, the reduction in untranscribed rDNA copies have been observed to increase the sensitivity to DNA damage in yeast (44). Given that preserving genomic integrity is a top priority for germ cells, and these cells have a low tolerance for DNA damage (46), it is likely that the germline would be particularly sensitive to reductions in rDNA CN. Furthermore, it is unclear what genetic or environmental factors influence the necessary number of untranscribed rDNA copies, and variation in the demand for surplus rDNA may contribute to the large variation in rDNA CN between Drosophila strains. Such deviations in rDNA CN requirements may underlie the inconsistency in the correlation between rDNA CN and phenotypic effect that we observed between experimental conditions. Future investigation into the direct causes of rDNA CN reduction to disrupt GSC physiology, in particular the roles of rRNA synthesis and transcription–replication collisions, is critical to fully understand the selective forces that impact the interaction between R2 and its host genome.

The widespread presence of R2 and other rDNA-specific TEs in both vertebrates and invertebrates (17) suggests that similar host–TE mutualism may support rDNA CN maintenance throughout Metazoa. Interestingly, many of the rDNA-specific TEs have little sequence similarity to R2, instead appearing to be derived from other nonspecific TEs (17), suggesting this host–TE mutualism may have evolved multiple times over the course of evolution. In summary, our study provides an example of mutualistic retrotransposons in the maintenance of eukaryotic genomes, and we propose that such host–TE relationships may be widespread throughout eukaryotes.

## Materials and Methods

### Immunofluorescence.

Immunofluorescence staining of testes was performed as previously described (47). Briefly, testes were dissected in PBS, fixed in 4% formaldehyde in PBS for 30 min, then briefly washed two times in PBS containing 0.1% Triton-X (PBS-T), followed by washing in PBS-T for 30 min. After washes, samples were incubated at 4 **°**C overnight with primary antibody in 3% bovine serum albumin (BSA) in PBS-T. Samples were washed three consecutive times for 20 min in PBS-T, then incubated at 4 **°**C overnight with secondary antibody in 3% BSA in PBS-T, washed three times again in PBS-T for 20 min, and mounted in VECTASHIELD with DAPI (Vector Labs). The following primary antibodies were used: rat antivasa (1:20; DSHB; developed by A. Spradling), mouse anti-Fascillin III (1:200; DSHB; developed by C. Goodman), and rabbit anti-γ-H2AvD pS137 (1:200; Rockland). Images were taken with a Leica Stellaris 8 confocal microscope with 63× oil-immersion objectives and processed using Fiji (ImageJ) software.

### RNA FISH and Image Quantification.

RNA FISH samples were prepared as previously described (7). In short, dissected testes were fixed in 4% formaldehyde in PBS for 30 min, briefly washed in PBS, and permeabilized in 70% ethanol overnight at 4°. Samples were then briefly rinsed in 2× SSC with 10% formamide prior to hybridization with 50 nM probes overnight at 37°. Samples were washed twice in 2× SSC with 10% formamide for 30 min and mounted in VECTASHIELD with DAPI (Vector Labs). Samples were imaged using a Leica Stellaris 8 confocal microscope with 63× oil-immersion objectives and processed using Fiji (ImageJ) software. R2 Stellaris FISH probe set was designed and synthesized by Biosearch Technologies. ITS probe sequence is listed in *SI Appendix*, Table S1.

For ITS RNA FISH signal quantification, nonsaturating images were taken of optimized z-slices throughout each imaged cell. ITS signal intensity was quantified using Fiji (ImageJ) software and signal intensity was summed across all z-slices for each cell. The summed intensity of each GSC was normalized to the summed intensity of a somatic Cyst Stem Cell within similar z planes. Only GSCs with a suitable normalizing Cyst Stem Cell within similar z planes were scored.

### DNA Isolation.

Testis DNA was isolated from 50 pooled dissected testes frozen in liquid N2. DNA isolation was performed according to previously described methods for isolation from Drosophila tissues (48). DNA was isolated from individual Drosophila animals using a modified protocol of the DNeasy Blood and Tissue DNA extraction kit (Qiagen). In short, individual animals were homogenized in 200 µL Buffer ATL containing proteinase K using a pipette tip in Eppendorf tubes, vortexed for 15 s, and incubated for 1.5 h at 56°. Samples were then prepared following the manufacturer’s protocol after incubation. All DNA samples were quantified and checked for purity by NanoDrop One spectrophotometer (ThermoFisher).

### rDNA Copy Number Measurement by Droplet Digital PCR (ddPCR).

Thirty nanograms of DNA sample were used per 20 µL ddPCR for control gene reactions (RpL and Upf1), and 0.3 ng of DNA per 20 µL ddPCR for 28S rDNA reactions. Primers and probes for reactions are listed at *SI Appendix*, Table S1. ddPCR were carried out according to the manufacturer’s (Bio-Rad) protocol. In short, master mixes containing ddPCR Supermix for Probes (No dUTP) (Bio-Rad), DNA samples, primer/probe mixes, and HindIII-HF restriction enzyme (New England Biolabs) for 28S rDNA reactions (no restriction enzyme for control gene reactions) were prepared in 0.2-mL Eppendorf tubes, and incubated at room temperature for 15 min to allow for restriction enzyme digestion. ddPCR droplets were generated from samples using QX200 Droplet Generator (Bio-Rad) and underwent complete PCR cycling on a C100 deep-well thermocycler (Bio-Rad). Droplet fluorescence was read using the QX200 Droplet Reader (Bio-Rad). Sample copy number was determined using Quantasoft software (Bio-Rad). rDNA copy number per genome was determined by 28S sample copy number multiplied by 100 (due to the 100× dilution of sample in the 28S reaction compared to control reaction) divided by control gene copy number multiplied by the expected number of control gene copies per genome (2 for RpL in all samples; 2 for Upf1 in female samples; 1 for Upf1 in male samples). The 28S copy number values determined by each control gene was averaged to determine 28S copy number for each sample.

### RNA Isolation.

Fifty dissected testes were pooled and frozen in liquid N2 for each RNA isolation sample. Samples were homogenized in 400 µL TRIzol™ (ThermoFisher Scientific) and RNA was isolated using Direct-zol™ RNA Miniprep kit (Zymo Research) according to manufacturer directions, including on-column DNase I treatment. All RNA samples were quantified and checked for purity by Nanodrop One spectrophotometer (ThermoFisher).

### Quantification of R2 Expression by Reverse Transcriptase (RT)-ddPCR.

Approximately 20 ng of total RNA was used per 20 µL RT-ddPCR for R2 reactions, and 0.2 ng of total RNA were used per 20 µL control gene (Tubulin) reaction. Tubulin primers and probe are listed at *SI Appendix*, Table S1, and R2 primers and probe mix were designed by Bio-Rad (Assay ID: dCNS858096478). ddPCR droplets were generated from samples using QX200 Droplet Generator (Bio-Rad) and underwent RT-PCR and endpoint PCR on a C100 deep-well thermocycler (Bio-Rad). Droplet fluorescence was read using the QX200 Droplet Reader (Bio-Rad). RNA quantitation was determined using QuantaSoft software (Bio-Rad), and R2 counts were normalized to Tubulin concentration for all samples. Normalized R2 expression values were then set relative to the average R2 expression value in control conditions.

### Quantification of ETS Expression by qRT-PCR.

Approximately 1 μg of total RNA was used for cDNA synthesis via SuperScript III First-Strand Synthesis (Invitrogen) using random hexamer primers, according to manufacturer directions. Real-time PCR was done using SYBR® Green PCR Master Mix (Applied Biosystems) and assessed with a QuantStudio 6 Flex system (Applied Biosystems). ETS expression values were normalized to GAPDH. Primers used are listed in *SI Appendix*, Table S1.

### Mitotic and Meiotic Chromosome Spread, DNA FISH, and Quantification.

Mitotic chromosome spreads in neuroblast cells, DNA FISH, and imaging were all done as previously described (49). In short, brains were dissected from male third instar larvae in PBS and fixed in 25 µL of acetic acid and 4% formaldehyde in PBS. Samples were applied to Superfrost plus slides and manually squashed under a coverslip, then immediately frozen in liquid N2. After freezing slides were removed from N2, coverslip removed, and slides were dehydrated in 100% ethanol and dried at room temperature. DNA FISH hybridization was performed in 20 µL of 50% formamide, 10% dextran sulfate, 2× SSC buffer and 0.5 µM each probe applied directly to the sample on the slide and covered with a cover slip. Samples were incubated at 95° for 5 min, cooled and wrapped in parafilm, then incubated overnight at room temperature in a dark humid chamber. Coverslips were removed and slides were washed three times for 15 min in 0.1× SSC, dried, and then mounted in VECTASHIELD with DAPI (Vector Labs). Samples were imaged using a Leica Stellaris 8 confocal microscope with 63× oil-immersion objectives and processed using Fiji (ImageJ) software. Meiotic chromosome spreads were prepared from dissected testes and imaged in the same manner. Relative Y:X rDNA fluorescence quantification was determined as previously described (7). Probes used for this study are as follows: 359, 5′-AGGATTTAGGGAAATTAATTTTTGGATCAATTTTCGCATTTTTTGTAAG-3′-Cy5; (TAGA)_6_-Cy5; IGS, 5′-AGTGAAAAATGTTGAAATATTCCCATATTCTCTAAGTATTATAGAGAAAAGCCATTTTAGTGAATGGA-3′-Alexa488; (AATAC)_6_-Cy3; and (AATAAAC)_6_-Cy3.

### Generation of rDNA Deletion Animals.

rDNA copy number loss was induced during larval development in *yw*/Y; *HS-I-CreI*, *Sb*/TM6B males with a *y, w* X chromosome by I-CreI expression as previously described (26). In brief, parental animals mated and laid eggs for 3 d, then removed from food. After one additional day of larval development, animals were exposed to 37 °C heat shock for 45 min on two consecutive days. To identify X chromosomes with significant rDNA copy number reduction (bb), adult males that experienced I-CreI expression were mated to *bb^158^*/*FM6* females, and virgin non-*FM6* daughters (bb/bb^158^) were screened for the *bobbed* phenotype. 28 out of 946 non-*FM6* daughters screened were *bobbed*. To isolate potentially reduced rDNA loci and remove HS-I-CreI from the background, TM6B containing *bobbed* females were individually mated to wildtype males. Male offspring from each individual female candidates were subsequently individually mated to *bb^158^*/*FM6* females. Any mating that failed to produce non-FM6 daughters were eliminated (due to having the bb^158^ and not the candidate bb chromosome). All viable non-FM6 daughters were double-checked for the *bobbed* phenotype, and stocks with all *bobbed* non-FM6 daughters had FM6 containing siblings collected and used to establish a bb/FM6 stock. This method isolated the novel rDNA deletion allele, bb^Z9^, used in this study.

### Drosophila Genetics.

All Drosophila lines used in this study are found in *SI Appendix*, Table S2. All animals we reared on standard Bloomington medium at 25°. All aging was done in roughly 1:1 mixed presence of males and females, provided fresh food every 4 to 6 d. *UAS-R2 RNAi* strains were designed using SNAP-DRAGON shRNA target software, and oligos containing target hairpin sequence were cloned into the WALLIUM20 vector for phiC31 site-directed integration into the Drosophila genome for expression of a short hairpin to create endogenous miRNAs (50). The target sequence for the *R2i-1* construct is 1481-CCGGTTGAACTCATCAATCAA-1502. The target sequence for the *R2i-2* construct is 432-CCAGACGAACTTGATGAAGAA-453. All UAS-R2 transgenes were synthesized into pUAST:attB by VectorBuilder (Chicago, IL) for site-directed integration. Importantly, these target sequences were specifically designed to target the R2 ORF encoding the R2 retrotransposase. ORF-containing R2 mRNA are expected to be translated in the cytoplasm, where it is subjected to silencing by the canonical RNAi mechanism. The UAS-R2 transgene contains the R2 ORF tagged with 3xFLAG tag at N terminus, cloned into the pUAST:attB vector. The UAS-R2 transgene also contains sense mutations at the *R2i-1* target sequence to render the transgene insensitive to this RNAi (1481-CCGGTTGAACTCATCAATCAA-1502 to 1481-ACGTCTTAATAGCAGTATTAA-1502. The Nuclease-Dead UAS-R2 transgene is identical to the UAS-R2 transgene except for 3001-AAACCAGAC-3009 to GCC and 3097-AAAATCAATAGA-3108 to 3097-GCCATCAATGCC-3108, which are analogous mutations to those demonstrated to disrupt *B. mori* R2 endonucelase activity (51). All injections and selection of animals containing integrated transgenes were performed by BestGene, Inc (Chino Hills, CA).

### rDNA Magnification and Heritability Assays.

Males containing the *bb^Z9^* allele were mated in bulk to *bb^158^*/*FM6*, *Bar* females. *bb^Z9^*/*bb^158^* female offspring were selected based on the absence of the *Bar* dominant marker, and scored for cuticular phenotype. To determine the frequency of heritability of magnified offspring, unmated *bb^Z9^*/*bb^158^* female F1 animals with wild-type cuticles were collected and individually mated with 3 *bb^158^*/Y males. Since homozygous *bb^158^* animals are lethal, all viable female F2 animals are *bb^Z9^*/*bb^158^* and were scored for cuticular phenotype. Each individual F1 animal was scored for their ability to produce any offspring with wildtype cuticles, and for the percentage of their F2 female offspring to have wildtype cuticles.

### Lineage Survival Assay.

Independent lineages of *nos-gal4*/*CyO*; *UAS-R2 RNAi*/*Tm6B* or *nos-gal4*/*CyO*; *TM2*/*TM6B* animals were established by collecting siblings of the indicated genotypes from *nos-Gal4*/*CyO*;*TM2*/*TM6B* males mated to *Sp*/*CyO*;*UAS-R2 RNAi*/*TM6B* females. At each generation in each lineage, three males were mated with five females for 5 d, and offspring were collected 10 d after mated ended. Any lineages that did not have at least three males and five females at collection time were terminated due to insufficient animals.

### Statistics.

For all comparisons of percentage of samples with categorical values (percent γH2Av or R2 positive cells), significance was determined by chi-squared test, and error bars were generated using the Confidence Interval for a Population Proportion formula. For all comparisons of samples with independent values (number of GSCs; rDNA copy number), significance was determined by Student’s *t* test between experimental and control conditions, unless otherwise indicated.

## Supplementary Material

Appendix 01 (PDF)

## Data Availability

All study data are included in the article and/or *SI Appendix*.
