# Supplementary material for: The retrotransposon R2 maintains Drosophila ribosomal DNA repeats
Source: Proc Natl Acad Sci U S A. 2023 May 30;120(23):e2221613120. doi: 10.1073/pnas.2221613120 (PMC10266012; doi:10.1073/pnas.2221613120)
Supplement: Supplementary file 1 — Appendix 01 (PDF) [file pnas.2221613120.sapp.pdf]

Supporting Information

Fig S1 – S8

Table S1 – S2

References 52 - 55

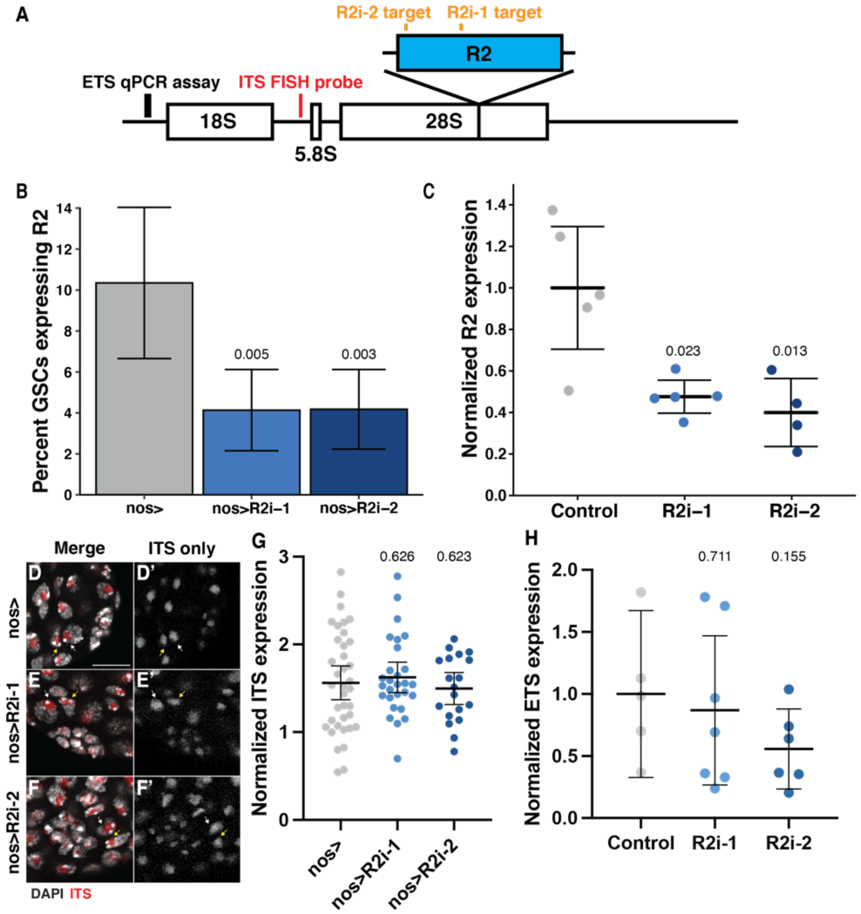

Figure S1: Design, efficiency, and specificity of R2 RNAi lines. **(A)** Diagram the 45S rRNA cistron constituting an 'rDNA copy' and the sequence-specific R2 insertion site in the 28S rRNA gene. Location of R2 RNAi target sequences indicated in orange. R2 ORF in blue rectangle, R2 UTRs as lines. External transcribed spacer (ETS) qPCR assay region in black. Internal transcribed spacer (ITS) FISH probe region in red. **(B)** Precent of GSCs expressing R2 detected by RNA FISH in *nos-gal4* driver only condition (*nos*>) and *nos-gal4*-driven R2 RNAi (*nos>UAS-R2i*). Two independent R2 RNAi constructs were used throughout the study. P-value determined by chi-squared test. Error bars = 95% CI. **(C)** Efficiency of R2 knockdown used in this study determined by RT-ddPCR, normalized to Tubulin and set relative to control expression. GSCs were enriched by co-expressing *upd* (52, 53), comparing *nos>upd* vs. *nos>upd, R2i*. P-value determined by Student's t-test. Error bars = 95% CI. **(D-F)** Images of ITS RNA FISH in germline stem cells (white arrow heads) and somatic cyst cells (yellow arrow heads). DAPI in white, ITS

in red. ITS only channel in D’-F’. **(G)** ITS expression in GSCs normalized to ITS expression in nearby cyst stem cells. P-value determined by Student’s t-test. Error bars = 95% CI. **(H)** ETS qPCR in *nos>upd* GSC-enriched testes normalized to GAPDH expression. P-value determined by Student’s t-test. Error bars = 95% CI.

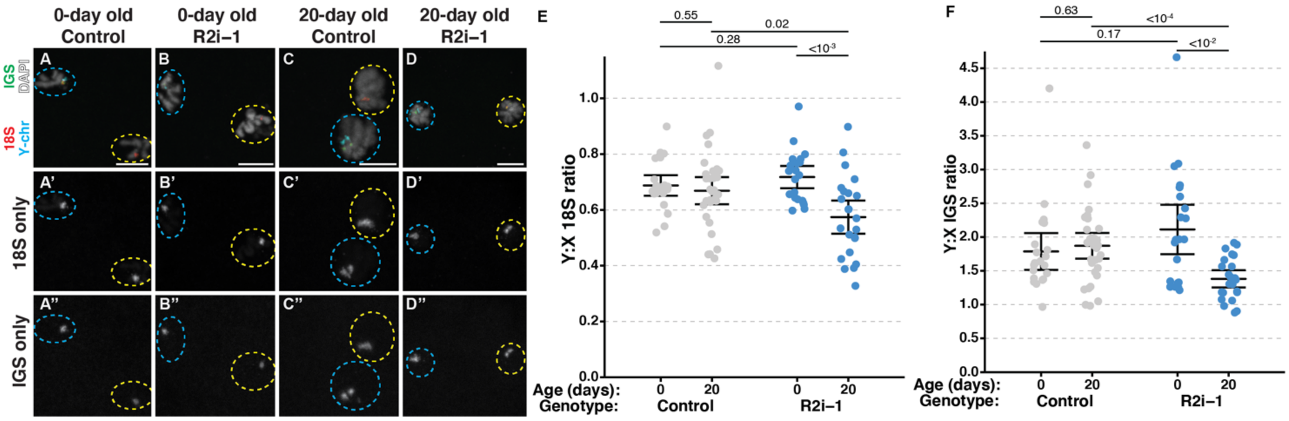

Figure S2: Germline R2 inhibition causes rDNA CN loss in germ cells during aging. (A-D) DNA FISH with 18S rDNA (Red), intergenic spacer (IGS) (Green) and the Y-specific AATAAAC (Cyan) probes on meiotic chromosome spreads from spermatocytes. Germline rDNA copy number loss during aging is biased on the Y chromosome, and thus results in a reduction in the relative Y:X ratio in rDNA (18S and IGS) FISH signal intensity (*5*). DAPI is shown in white. Y-containing chromatids are indicated by cyan dotted circle, X-containing chromatids are indicated by yellow dotted circle. A’-D’: 18S (red channel), and A’’-D’’ IGS (green channel). Scale bar = 5 μm. **(E)** Relative Y:X 18S rDNA ratio of DNA FISH signal intensity. P-value determined by Student’s t-test. Error = 95% CI from the mean. **(F)** Relative Y:X IGS ratio of DNA FISH signal intensity. P-value determined by Student’s t-test. Error = 95% CI from the mean.

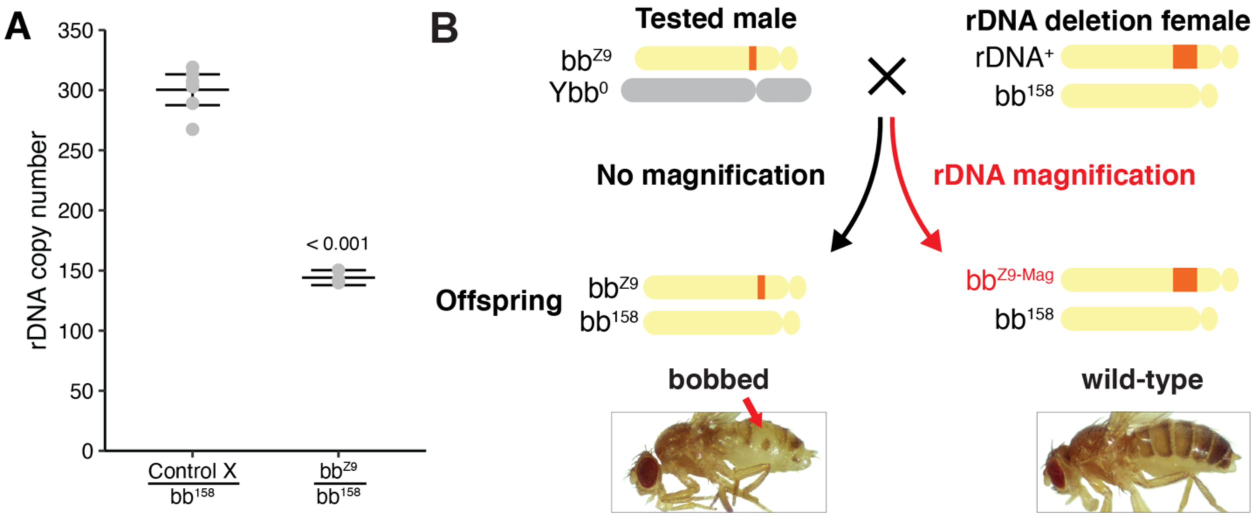

Figure S3: Diagram of phenotypic assessment to detect rDNA magnification at the bb^{Z9} allele. (A) rDNA CN quantification of the bb^{Z9} allele determined by ddPCR. All samples from individual females with either the bb^{Z9} allele or X chromosome from which the allele was isolated heterozygous with an X chromosome completely lacking rDNA (bb^{158}). P-value determined by Student's t-test. Error bars = 95% CI. **(B)** Scheme to detect rDNA magnification of bb^{Z9} allele. Tested magnifying males (bb^{Z9} / Ybb^{0}) are crossed to females heterozygous for the bb^{158} rDNA deletion allele. Resultant bb^{Z9} / bb^{158} daughters completely rely on the bb^{Z9} locus for their rDNA. (Left) bb^{Z9} / bb^{158} offspring with the 'bobbed' cuticular defects associated with rDNA insufficiency (red arrow) are determined to have inherited a bb^{Z9} allele that did not undergo rDNA magnification. (Right) bb^{Z9} / bb^{158} offspring with wild-type cuticles are determined to have inherited a bb^{Z9} allele that restored normal rDNA content through rDNA magnification ($bb^{Z9\text{-}Mag}$). Thus, the portion of bb^{Z9} / bb^{158} offspring with wild-type cuticles represents the portion of bb^{Z9} alleles that underwent rDNA magnification. bb^{Z9} / bb^{158} animals are also collected for rDNA CN quantification, and total rDNA CN in these animals represents the rDNA from the bb^{Z9} locus alone.

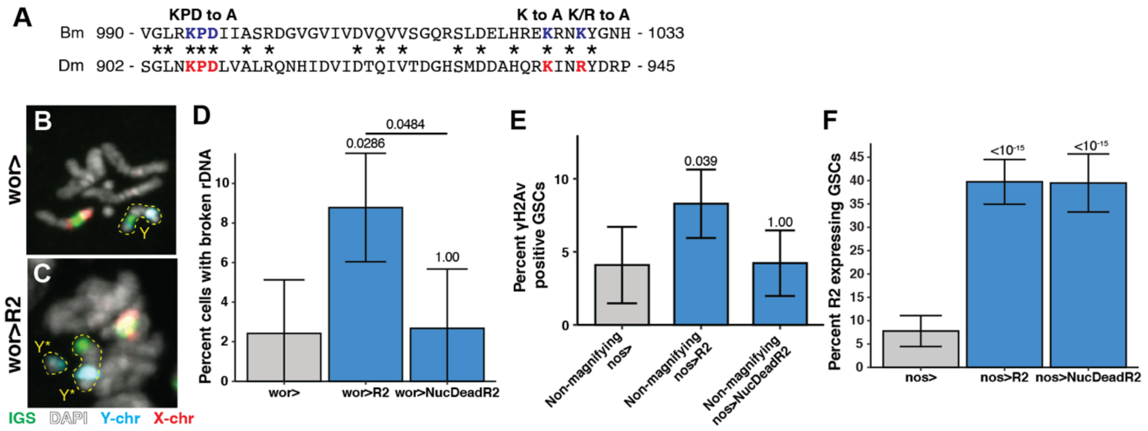

Figure S4: Transgenic R2 expression induces double stranded breaks at rDNA loci. (A) Alignment of 44 amino acids in the endonuclease domain of *Bombyx mori* R2 (Bm) and *Drosophila melanogaster* R2 (Dm). Residues shown to be essential for Bm R2 endonuclease activity(6) labeled in blue and respective Dm R2 residues in red. Mutations found to render Bm R2 endonuclease dead(6) and their respective amino acid changes in Dm R2 are indicated above highlighted residues. **(B-C)** DNA FISH in mitotic chromosome spreads of larval neuroblasts in animals with the neuroblast driver *wor-gal4* (wor>, B) or transgenic R2 expressed by the *wor-gal4* driver (wor>*R2*, C) animals. rDNA is labelled by probes targeting the rDNA intergenic sequence (IGS) (Green), Y- chromosome is labelled by probes targeting AATAC and AATAAAC satellite sequences (Cyan) and X-chromosome is labelled by probes against 359 and TAGA satellite sequences (Red). Y* indicates Y-chromosome fragments broken at the rDNA locus. **(D)** Percentage of neuroblasts with DSBs at rDNA loci determined by DNA FISH in mitotic spreads in indicated genotypes. P-values determined by chi-squared test. Error = 95% CI. **(E)** Percentage of γH2Av positive GSCs in the indicated genotypes. Transgenic R2 constructs expressed in the germline by the *nos-gal4* driver (*nos*>). P-value determined by chi-squared test. Error = 95% CI. **(F)** Percentage of GSCs expressing R2 determined by RNA FISH in control and R2 over-expression conditions. P-values determined by chi-squared test. Error = 95% CI.

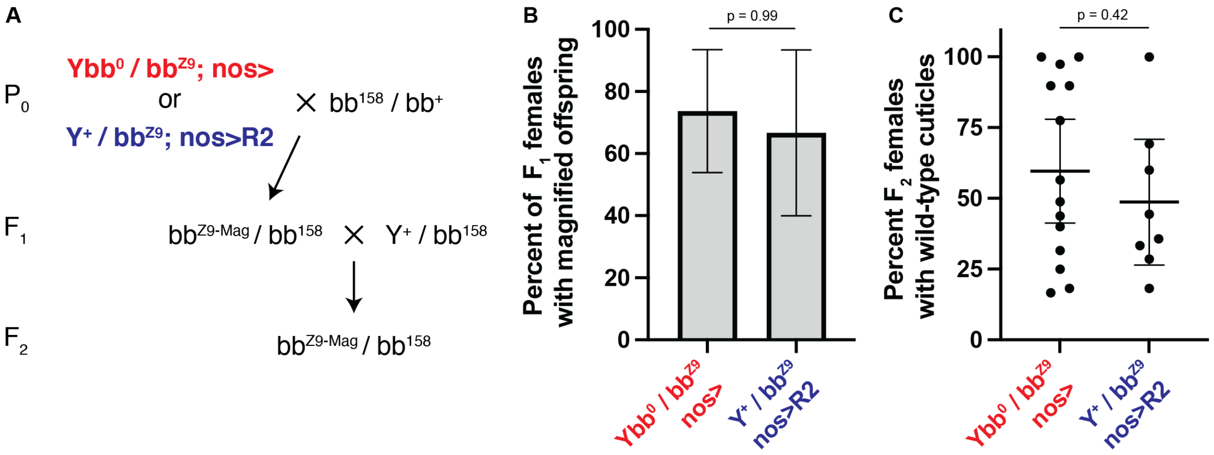

Figure S5: rDNA magnification induced by insufficient rDNA copy number (*Ybb/bb*^{z9}) or ectopic R2 expression is stably inherited. (A) Scheme to assess stable inheritance of rDNA magnification. P_0 Males capable of rDNA magnification (*Ybb*^{0} */ bb*^{Z9}*; nos> and Y*$^{+}$ */ bb*^{Z9}; *nos>R2*) were mated to females heterozygous for a complete X-chromosome rDNA deletion (bb^{158}). Resultant putatively magnified *bb*$^{Z9\text{-}Mag}$ */ bb*^{158} F_1 daughters with wild-type cuticles were mated with *bb*^{158}*/Y*$^{+}$ males, and the F_2 daughters *bb*$^{Z9\text{-}Mag}$ */bb*^{158} were examined for their cuticle phenotype. F_1 animals that produced *bb*$^{Z9\text{-}Mag}$ */ bb*^{158} F_2 daughters with wild-type cuticles were determined to have stably inherited rDNA magnification. **(B)** Frequency of F_1 females from indicated P_0 males that produced any F_2 animals with wild-type cuticles. Error = 95% CI. P-value by Chi-squared test. **(C)** Frequency of F_2 *bb*$^{Z9\text{-}Mag}$ */ bb*^{158} offspring that have wild-type cuticles from stably magnified F_1 daughters from P_0 males of the indicated genotypes. The incomplete penetrance of wild-type cuticles among heritable rDNA magnification suggests epigenetic factors can influence the cuticular phenotype in animals with sub-optimal rDNA CN. Error = 95% CI. P-value determined by Student's t-test.

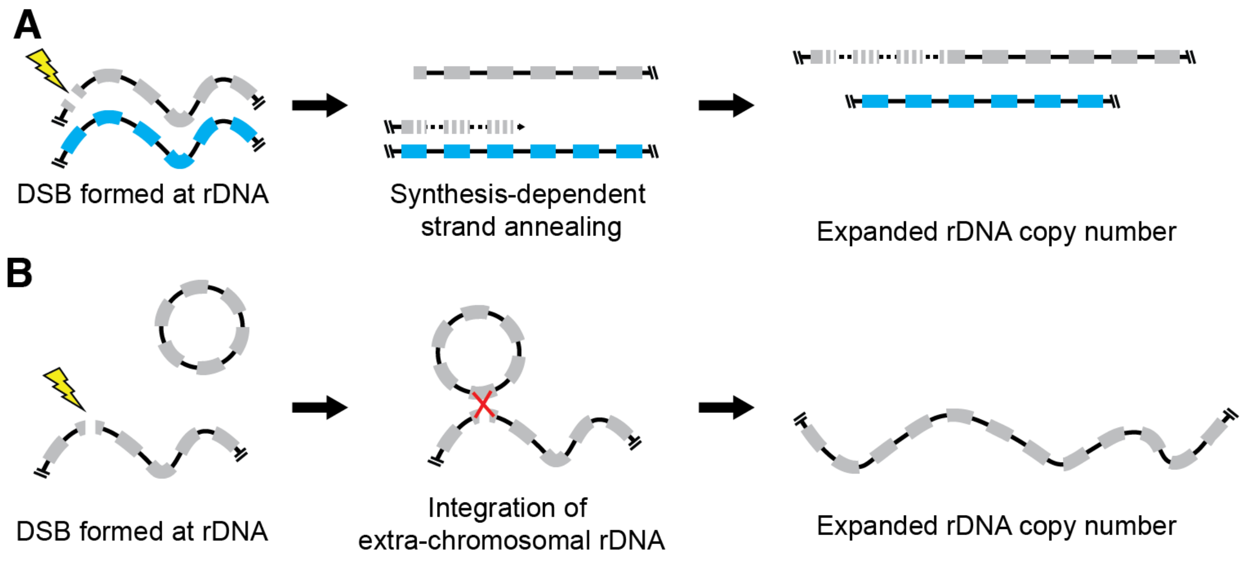

Figure S6: Models of rDNA CN expansion initiated by DSBs at rDNA locus. (A) rDNA CN expansion by strand-dependent strand annealing (SDSA). DSBs at rDNA loci can be repaired via recombination of a single strand with sister chromatid and synthesis. Synthesis can run on to replicate multiple rDNA copies before re-annealing with broken strand. Upon repair after synthesis, rDNA CN is expanded on one sister chromatid while CN of the other sister remains intact. **(B)** DSBs formed at rDNA loci induce homology-dependent repair that causes recombination with existing extrachromosomal rDNA circles (ERCs), allowing for reintegration of the ERC rDNA copies into the genomic rDNA locus.

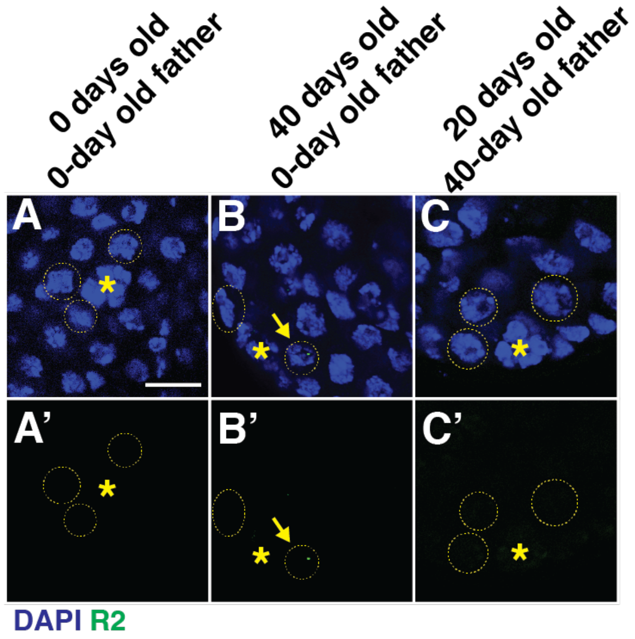

Figure S7: R2 expression in GSCs is regulated in response to changing rDNA copy number during aging. (A-C) R2 RNA FISH in the male germline of young (0-days old), (A) old (40-days old) (B), and recovered offspring of old animals (C). Isolated R2 channel in A’-C’. GSCs indicated by yellow dotted circle. * indicates GSC niche signal hub. R2 positive cells indicated by yellow arrowhead.

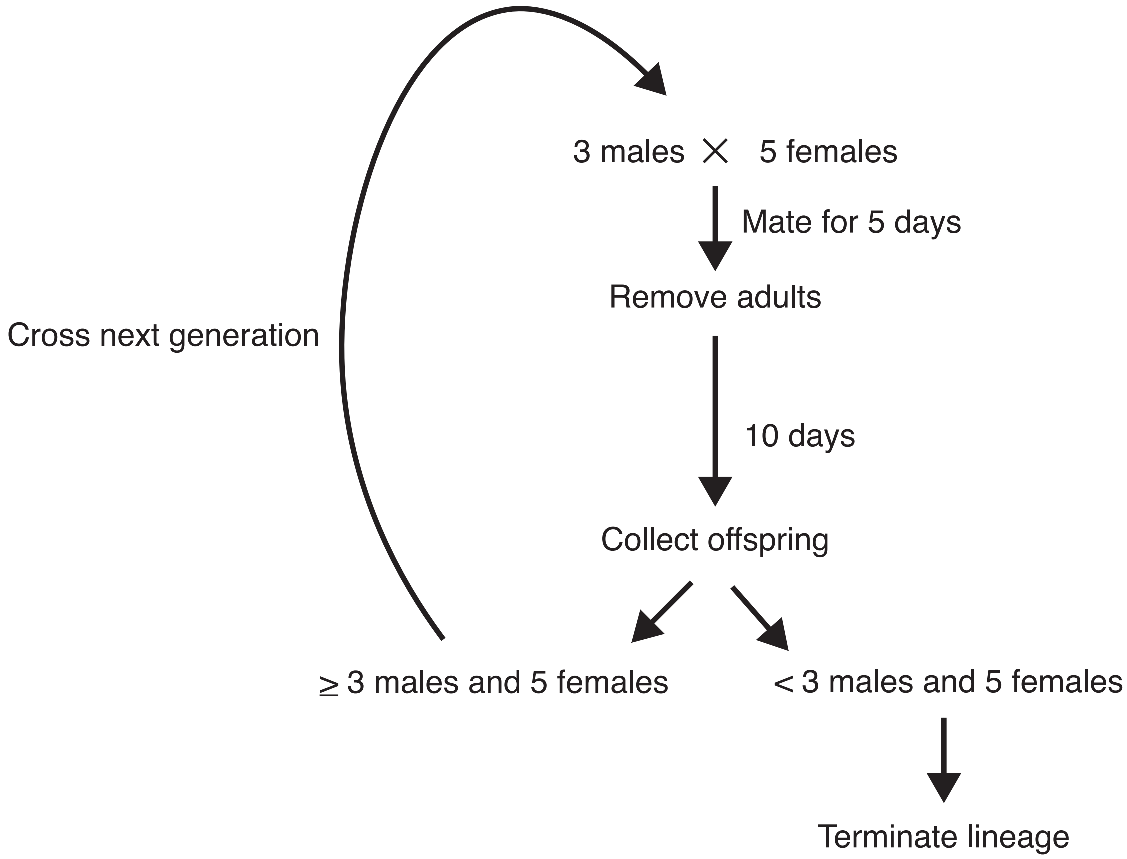

Figure S8: Scheme for testing lineage survival in multi-generational R2 RNAi experiment. Each lineage is established using 3 males and 5 females of the given genotype. Males and females are mated for 5 days and removed from the vial. 10 days later, all offspring are collected. If total offspring from the mating contains at least 3 males and 5 females, then 3 males and 5 females are randomly selected to establish the next generation of the lineage. If the total offspring contained fewer than 3 males or 5 females, then the lineage was terminated and future generations were not established. This procedure was repeated at each generation for each lineage for 6 generations or until termination.

Table S1: List of oligomers used in this study for ddPCR, qPCR, and RNA FISH assays.

Oligo Name	Type	Target	Sequence	Modifications
dd-RpL32 F	Primer	RpL32	GCTTCAAGGGACAGTATCTG	
dd-RpL32 R	Primer	RpL32	AACGCGGTTCTGCATGAG	
dd-RpL32 Probe	Probe	RpL32	ATGCCCAACATCGGTTAC	5' HEX AND 3' Iowa Black FQ
dd-28S F	Primer	28S	GAGCTGCCATTGGTACAG	
dd-28S R	Primer	28S	GCTTTCGCCTTGAACTTAG	
dd-28S Probe	Probe	28S	TGGTGGATAGTAGCAAATAATCG	5' 6-FAM AND 3' Iowa Black FQ
dd-Upf1 F	Primer	Upf1	CACACTTTATGTCCACCATTATTG	
dd-Upf1 R	Primer	Upf1	GAGTTTCCGTAGGGACCAC	
dd-Upf1 Probe	Probe	Upf1	CCGTAACCGCCACTGCGGT	5' 6-FAM AND 3' Iowa Black FQ
dd-Tubulin F	Primer	αTub84B	GAGCAGCTGATCACTGGTAAGG	
dd-Tubulin R	Primer	αTub84B	CGAGTGGAAGATGAGGAAGCCC	
dd-Tubulin Probe	Probe	αTub84B	CTGTCCAGAACCAGATCGACGATCTCCTTG	5' 6-FAM AND 3' Iowa Black FQ
qETS Forward	Primer	rDNA ETS	ATTACCTGCCTGTAAAGTTGG	
qETS Reverse	Primer	rDNA ETS	CCGAGCGCACATGATAATTCTTCC	
qGAPDH Forward	Primer	GAPDH	TAAATTCGACTCGACTCACGGT	
qGAPDH Reverse	Primer	GAPDH	CTCCACCACATACTCGGCTC	
ITS antisense	RNA FISH Probe	rDNA ITS	ATTAGCCAACGTATGCCCATAACTA	5' Cy5

Table S2: List of Drosophila stocks used in this study.

Genotype	Source	Use
Dp(1;f)122; C(1)RM, y^1 v^1 f^1 */ C(1;Y)6, Df(1)259,* w^1	Kyoto Drosophila Genomics and Genetic Resource Center (DGRC) #107274	Mini-x Chromosome (*Dp(1;f)*)
yw	Bloomington Stock Center (BSC) #1495	Source for isolating novel rDNA deletion strain
w[1118]; P{v[+t1.8]=hs-I-CreI.R}1A Sb[1]/TM6	BSC #6937	I-CreI endonucelase
nos-gal4	PMID: 9501989	Early germ cell driver (54)
wor-gal4	BSC #56553	Neuroblast driver
bam-gal4	PMID: 12571107	Germ cell driver (55)
y[1] eq[1]/Df(YS)bb[-]	DGRC #101260	Y chromosome complete rDNA deletion
Df(1)bb158, y[1] / Dp(1;Y)y[+] / C(1); ca[1] awd[K]*	DGRC #106876	X chromosome complete rDNA deletion
UAS-upd	PMID: 10346822	Upd over expression to create GSC enrichment in testis

Supplementary References

52. A. A. Kiger, D. L. Jones, C. Schulz, M. B. Rogers, M. T. Fuller, Stem Cell Self-Renewal Specified by JAK-STAT Activation in Response to a Support Cell Cue. *Science*. 294, 2542–2545 (2001).

53. N. Tulina, E. Matunis, Control of Stem Cell Self-Renewal in Drosophila Spermatogenesis by JAK-STAT Signaling. *Science*. 294, 2546–2549 (2001).

54. M. V. Doren, A. L. Williamson, R. Lehmann, Regulation of zygotic gene expression in Drosophila primordial germ cells. *Curr Biol*. 8, 243–246 (1998).

55. D. Chen, D. M. McKearin, A discrete transcriptional silencer in the bam gene determines asymmetric division of the Drosophila germline stem cell. *Development*. 130, 1159–1170 (2003).
